# Inducing Energetic Switching Using Klotho Improves Vascular Smooth Muscle Cell Phenotype

**DOI:** 10.3390/ijms23010217

**Published:** 2021-12-25

**Authors:** Craig K. Docherty, Anastasiya Strembitska, Christa P. Baker, Fiona F. Schmidt, Kieran Reay, John R. Mercer

**Affiliations:** Institute of Cardiovascular and Medical Sciences, College of Medical Veterinary and Life Sciences, University of Glasgow, University Avenue, Glasgow G12 8TA, UK; craig.docherty@glasgow.ac.uk (C.K.D.); anastasiya.strembitska@unil.ch (A.S.); cpbaker@dundee.ac.uk (C.P.B.); Fiona_schmidt@t-online.de (F.F.S.); 2264537r@student.gla.ac.uk (K.R.)

**Keywords:** mitochondrial ATP generation, mitochondrial dysfunction, vascular smooth muscle cells, oxidative metabolism, glycolysis

## Abstract

The cardiovascular disease of atherosclerosis is characterised by aged vascular smooth muscle cells and compromised cell survival. Analysis of human and murine plaques highlights markers of DNA damage such as p53, Ataxia telangiectasia mutated (ATM), and defects in mitochondrial oxidative metabolism as significant observations. The antiageing protein Klotho could prolong VSMC survival in the atherosclerotic plaque and delay the consequences of plaque rupture by improving VSMC phenotype to delay heart attacks and stroke. Comparing wild-type VSMCs from an ApoE model of atherosclerosis with a flox’d Pink1 knockout of inducible mitochondrial dysfunction we show WT Pink1 is essential for normal cell viability, while Klotho mediates energetic switching which may preserve cell survival. Methods: Wild-type ApoE VSMCs were screened to identify potential drug candidates that could improve longevity without inducing cytotoxicity. The central regulator of cell metabolism AMP Kinase was used as a readout of energy homeostasis. Functional energetic switching between oxidative and glycolytic metabolism was assessed using XF24 technology. Live cell imaging was then used as a functional readout for the WT drug response, compared with Pink1 (phosphatase-and-tensin-homolog (PTEN)-induced kinase-1) knockout cells. Results: Candidate drugs were assessed to induce pACC, pAMPK, and pLKB1 before selecting Klotho for its improved ability to perform energetic switching. Klotho mediated an inverse dose-dependent effect and was able to switch between oxidative and glycolytic metabolism. Klotho mediated improved glycolytic energetics in wild-type cells which were not present in Pink1 knockout cells that model mitochondrial dysfunction. Klotho improved WT cell survival and migration, increasing proliferation and decreasing necrosis independent of effects on apoptosis. Conclusions: Klotho plays an important role in VSMC energetics which requires Pink1 to mediate energetic switching between oxidative and glycolytic metabolism. Klotho improved VSMC phenotype and, if targeted to the plaque early in the disease, could be a useful strategy to delay the effects of plaque ageing and improve VSMC survival.

## 1. Introduction

Cardiovascular diseases such as atherosclerosis exhibit profound ageing of the vessel wall that is linked to excess DNA damage and mitochondrial dysfunction [1]. Indeed, vascular smooth muscle cells found in disease-prone regions are characterised by defects in DNA repair, oxidative metabolism, and shortened telomeres. Collectively, these then activate signalling pathways to mediate cell cycle arrest and apoptosis which promote cell senescence and inflammation [2,3]. During disease, there is significant heterogeneity in the wall’s response to this injury with differential expression of disease-associated genes that alter responses to cell adhesion, proliferation, and migration, with loss of extracellular components that promote plaque development and susceptibility to plaque rupture [4].

Aged VSMC mitochondria appear to lose their ability to generate energy-carrying adenosine triphosphate (ATP). Intervening in oxidative metabolism required for normal cellular homeostasis is an attractive candidate to improve plaque cellular energetics. Indeed, our previous work has shown that diseased VSMCs retain their ability to energetically switch between oxidative and glycolytic metabolism. However, to date, there has been no effort to identify potential drug candidates that may mediate this switch. If a regulatable switch to aerobic glycolysis could be achieved pharmacologically, then improving VSMC survival could be a realistic goal.

Klotho is a powerful antiageing hormone whose abundance naturally decreases with normal human ageing [5]. Early transgenic studies show that while its knockout accelerates age-related disorders, overexpression extended lifespan in mice [6] and its supplementation increases lifespan in mice [7]. The role of Klotho in cardiovascular disease has suggested that its deficiency correlates with coronary artery disease, atherosclerosis, myocardial infarction, and left ventricular hypertrophy, with low abundance considered as an early predictor of atherosclerosis, while its abundance in ageing is regarded as protective to disease. This is largely believed to be because Klotho acts as both an antioxidant, via the PI3K/Akt pathway and, subsequently, enhanced FoxO-mediated expression of manganese superoxide dismutase (MnSOD), as well as having shown a role in apoptosis [8].

Klotho exists in three isoforms of α, β, and γ which may be membrane bound, intracellular, and secreted. Klotho is found as both a receptor and cleavable ligand [7] and has been shown to mediate several important antiageing effects in disease. It naturally enters the circulation from arterial and renal sources and inhibits insulin signalling [9] by improving cellular metabolism by enhancing glycolysis [10]. Membrane-bound Klotho acts as a co-receptor for fibroblast growth factor receptor for FGF23 and regulates phosphate homeostasis. Intracellular Klotho is an anti-inflammatory factor and suppresses cytokine recruitment. While secreted Klotho is essential for protecting cells from oxidative stress, maintaining calcium homeostasis, and inhibiting IGF1 and TGF-β1 receptors [11,12]. In particular, it links several glucose-dependent pathways, including glucose uptake, insulin sensitivity, and glucose and glycogen synthesis that would be key in the context of energetic switching [13].

It also acts as glucuronidase and protects against endothelial dysfunction by regulating nitric oxide by impacting signalling pathways including the p53/p21 axis, cAMP, protein kinase C, and the Wnt signalling pathways [14], all important to the atherosclerosis phenotype.

Building upon our previous work to characterise the role of Pink1-KO in atherosclerotic lesions, this study uses the generation of a vascular specific and inducible Pink1-KO cell line as a novel model of mitochondrial dysfunction [15]. Here, we investigate the next logical step to investigate if the Pink1 pathway can be augmented by potential drug candidates to mitigate defects in energetic homeostasis found in atherosclerotic development and plaque cap thinning. As we have previously shown, Pink1 is an important regulator of VSMC energetic switching from oxidative phosphorylation to glycolytic metabolism, and we now determined if the known energetic switching effects of Klotho were relevant in VSMCs and influenced by Pink1.

## 2. Results

### 2.1. Dose–Response Cytotoxicity Assay

**Figure 1:**
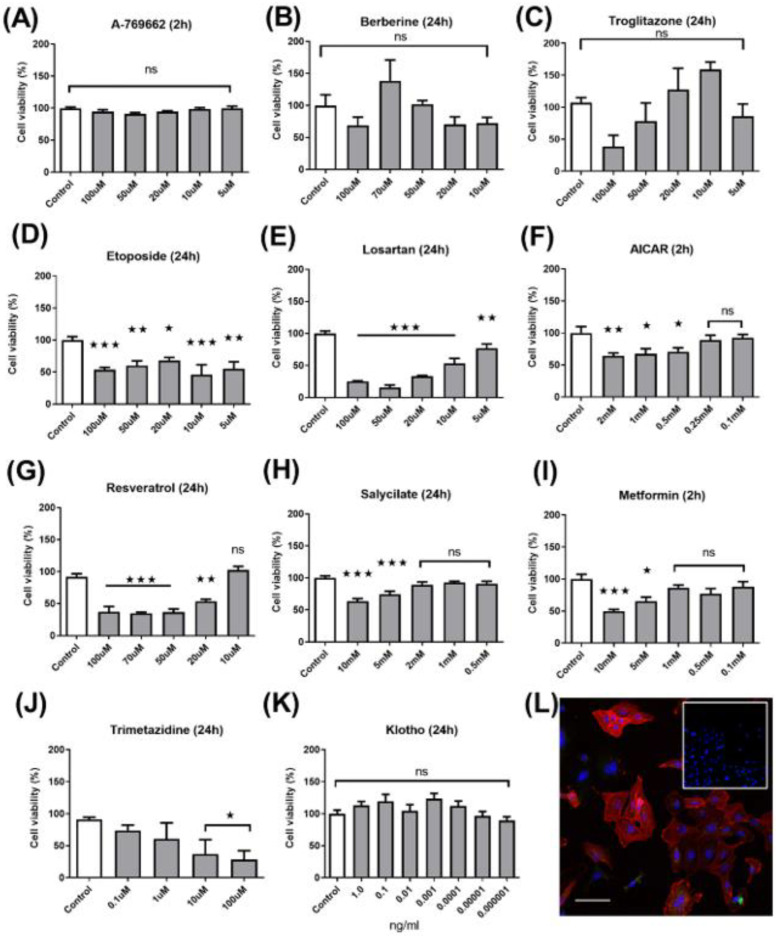
Cytotoxicity to screen acute and chronic dose-response. A screen of potential energetic switching compounds was performed. Both chronic (24 h) and acute (2 h) dosing regimens were tested in wild-type cells and rate of XTT conversion was used as an index of both mitochondrial health and viability. Survival was compared with no drug carrier controls: (**A**) A769662; (**B**) berberine; (**C**) troglitazone; (**D**) etoposide; (**E**) losartan; (**F**) AICAR; (**G**) resveratrol; (**H**) salicylate; (**I**) metformin; (**J**) trimetazidine; (**K**) Klotho; (**L**) confocal immunofluorescent imaging of the AMPK downstream marker acetyl–CoA carboxylase in explant VSMCs that are α-SMA positive (inset secondary antibody control) was used as a recognised surrogate marker of AMPK activity. (Scale 100 µM) and confirm VSMC status One-way ANOVA with Dunnett’s post hoc test (*n* = 4), * *p* ≤ 0.05, ** *p* ≤ 0.01, *** *p* ≤ 0.001.

We identified 10 compounds from the literature that have been shown to play important roles in cell survival, either through activation of the energy sensor AMPK or by evidence of inducing energetic switching between oxidative and glycolytic metabolism. To assess their suitability, a dose–response and cytotoxicity assay was performed (Figure 1A–K) with drug concentration ranges based on evidence from the literature. Explant cells were routinely assessed for VSMC markers including α-SMA, myosin heavy chain, vimentin, and calponin. In addition, confocal microscopy was used to assess the abundance of the AMPK downstream marker acetyl–CoA carboxylase (Figure 1L). While troglitazone showed a promising dose-dependent effect, this did not reach statistical significance. Equally, we excluded etoposide, losartan, AICAR, salicylate on the basis of substantial cytotoxicity. Notably, while the majority of experiments were performed after 24 h, those for A769662, AICAR, and metformin were performed at higher drug concentrations and shorter incubation of 2 h, based on prior experimental experience and evidence of off-target effects from the literature (Supplementary Table S1).

### 2.2. Protein Western Blot Analysis

**Figure 2:**
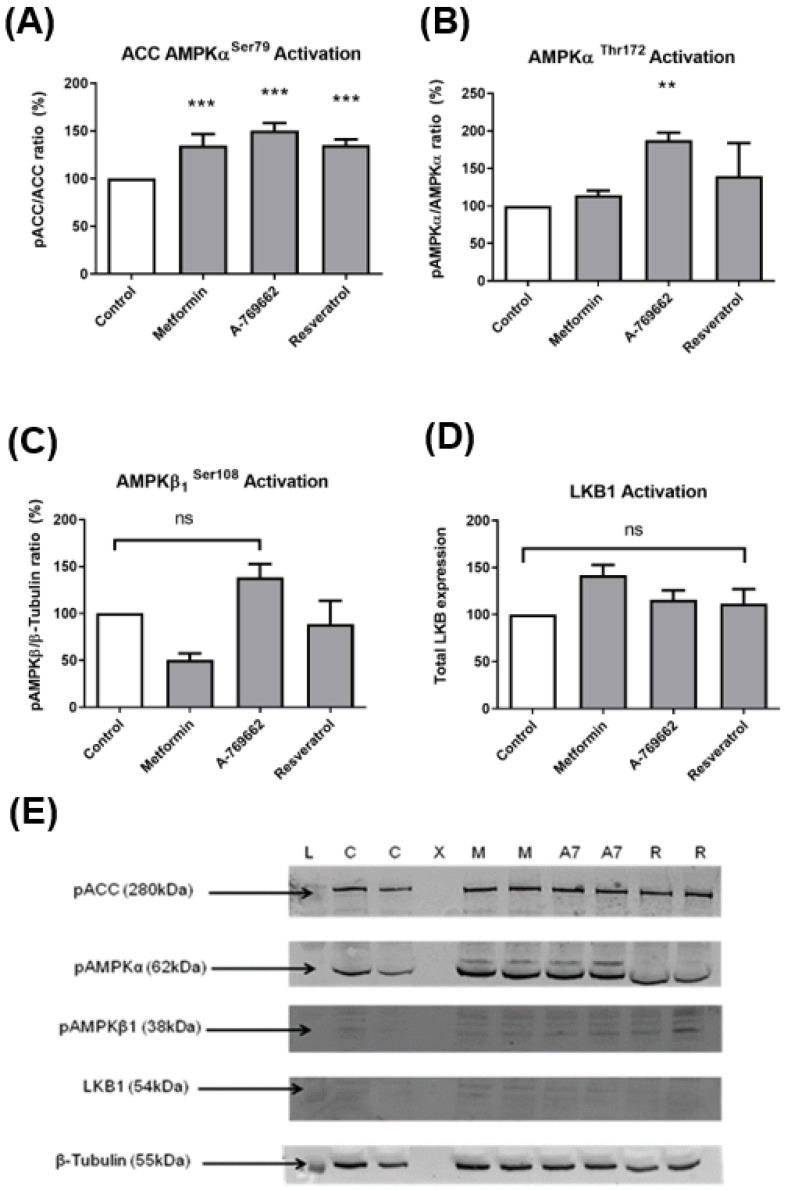
(**A**–**D**) Representative Western blot and quantification of pACC ser79, pAMPKα, pAMPKβ1, and LKB1 normalised to β-tubulin after a selection of drug treatments: protein ladder (L), no treatment control (C), blank X, metformin (M), A769662 (A7), resveratrol (R); (**E**) Western blots were quantified using LICOR technology (one-way ANOVA with Dunnett’s post hoc test (*n* = 4) ** *p* ≤ 0.01 vs. control. ** *p* ≤ 0.001 vs. control.

Metformin (0.1–1 mM), A769662 (5–100 µM), and resveratrol 10 µM were taken forward as potential candidate drugs for an AMPK activation assay using Western blot. (Figure 2A–E) (Supplemental Table S2). We used AMPKα activation by AMPK substrate (ACC) phosphorylation at serine 79 (Figure 2A), and AMPK threonine 172 (Figure 2B), AMPK β1 Serine 108 (Figure 2C), and the AMPK upstream kinase LKB1 by RT-qPCR (Figure 2D). Western blot data were normalised to β-tubulin. Despite initially promising results, only A-769662 showed efficacy in the activation of AMPK-α. However, when this candidate was taken through to validation in live-cell imaging, it performed poorly across a range of doses. Similar to A769662, Klotho was the only drug that appeared non-cytotoxic across the entire range of drug doses (Figure 1K). Therefore, as AMPK activation seemed an unreliable surrogate marker of drug efficacy, we proceeded to appraise its potential more directlyusing XF24 technology (Agilent, Santa Clara, CA, USA).

### 2.3. Energetic Analysis

**Figure 3:**
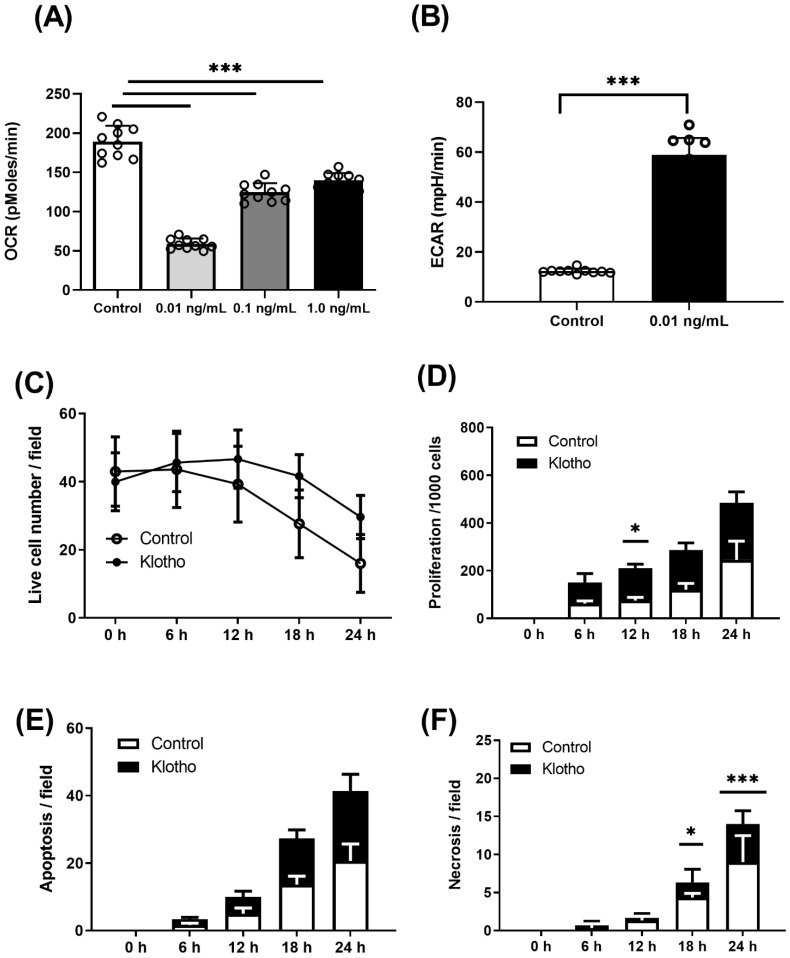
Plaque cell and tissue energetic analysis: (**A**) wild-type (Pink+/+) VSMC show decreased oxygen consumption rate (OCR) in response to Klotho and (**B**) concomitant energetic switching by upregulation of glycolysis (ECAR) in presence of Klotho; (**C**) no significant difference in survival between WT and Klotho-treated VSMCs; (**D**) rates of proliferation by completed mitosis events; (**E**) apoptosis rate over 24 h; (**F**) necrosis rate over 24 h; two-way ANOVA with Bonferroni’s post hoc test (*n* = 3–8), * *p* ≤ 0.05, *** *p* ≤0.001.

XF24 quantifies oxidative phosphorylation by measuring oxygen consumption rate (OCR) as a direct measure of complex 4 activity as the terminal electron acceptor of the mitochondrial respiratory chain, while simultaneously measuring extracellular acidification rate (ECAR) as cytoplasmic milli-pH as in index of glycolysis through glucose to lactate conversion. Drug doses for energetic assays were all 1 µM final concentration for mitochondrial inhibitors oligomycin, FCCP and rotenone, and myxothiazol, and 2-deoxy-glucose (25 mM) to competitively inhibit glucose (2.5 mM), as previously published [15]. In vivo, soluble Klotho is found to have a wide range of physiological doses (pg–ng) [16]. Therefore, in vitro where drug kinetics are substantially different, we assessed Klotho efficacy across the lowest reasonable doses (0.01–1.0 ng) (Figure 3A). We found 0.01 ng/mL Klotho showed the greatest effect on suppressing OCR and offering a commensurate switching effect by increasing ECAR (Figure 3B).

### 2.4. Live-Cell Imaging

To validate if these changes in metabolism lead to a functional change in cell behaviour, we performed live-cell imaging (Olympus BX51, Southend-on-Sea, United Kingdom, CellSenS). First, we compared the control wild-type VSMCs’ response in the presence and absence of Klotho, through which we observed improved cell survival (Figure 3C). We then assessed the effect of Klotho on the rate of proliferation across 24 h that reached significance but only at 12 h. In addition, we tracked single-cell responses using a scratch assay with Klotho, reducing the time taken for gap closure by improved cell migration over 24 h (Supplementary Figure S1A). In addition, it was observed that Klotho increased the rate of apoptosis (Figure 3E). Initially, we appraised the cell phenotypes by bright-field microscopy (Supplementary Figure S1B) and then used an enhanced method using a fluorescent triple dye (Ab142020, Abcam, UK), finding a large portion of this death could be categorised as necrotic after 18 and 24 h (Figure 3F).

**Figure 4:**
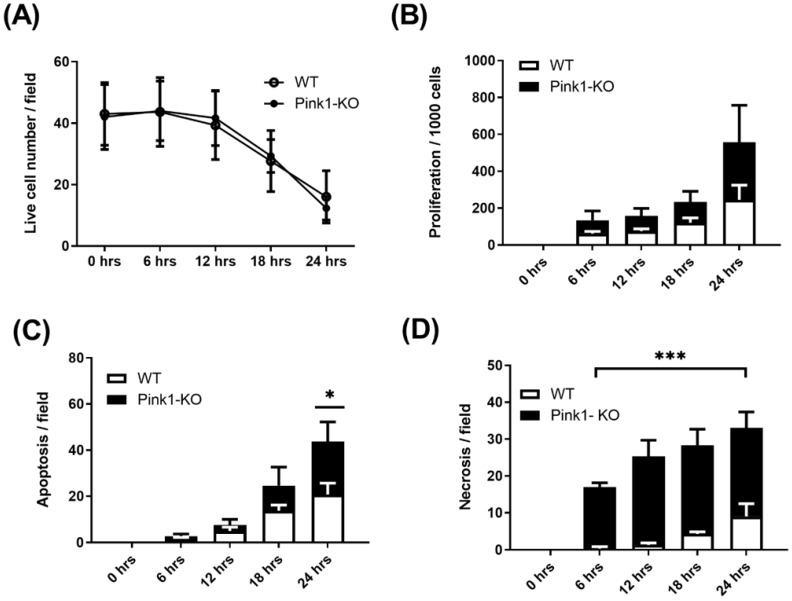
Live-cell comparison of wild-type (WT) and Pink1-KO VSMC: (**A**) wild-type survival, compared with Pink1-KO VSMC; (**B**) comparison of cell proliferation; (**C**) rates of apoptosis over 24 h; (**D**) rates of necrosis over 24 h. Two-way ANOVA with Bonferroni’s post hoc test (*n* = 3). * *p* ≤ 0.05, *** *p* ≤ 0.001.

**Figure 5:**
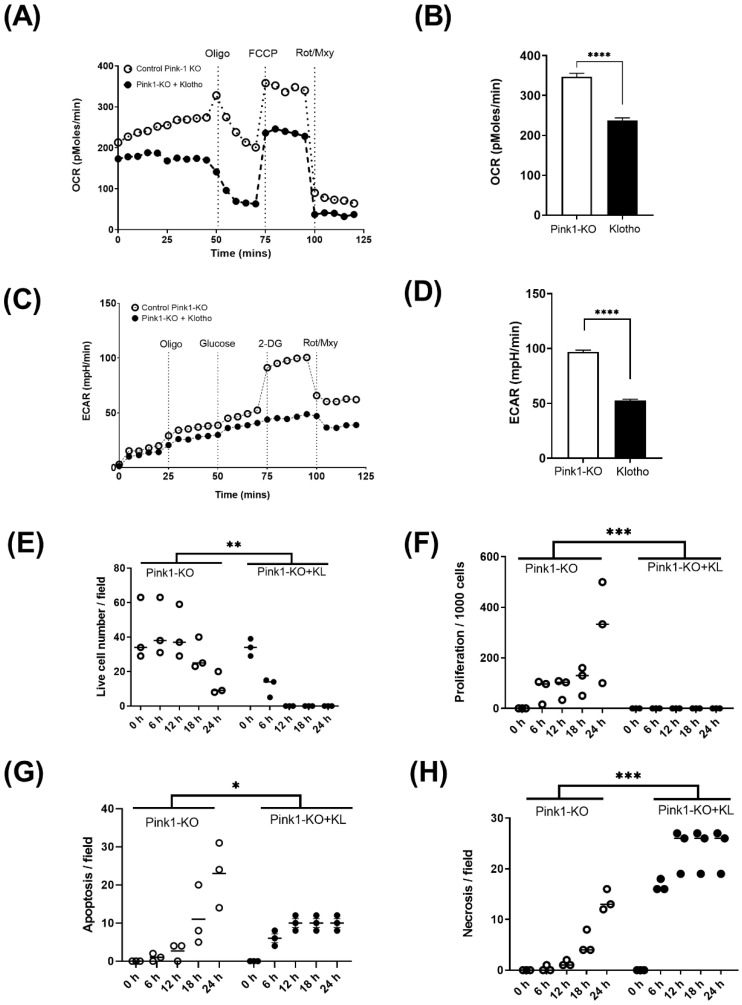
Pink-1 KO and Klotho energetic performance with live-cell analysis: (**A**) oxygen metabolism in Pink1-KO with Klotho; (**B**) total change in OCR; (**C**) Pink1-KO glycolytic metabolism with Klotho; (**D**) total change in ECAR; (**E**) Pink1-KO cell survival over 24 h in presence of Klotho; (**F**) Klotho-inhibited proliferation of Pink1-KO cells; (**G**) reduced apoptosis; (**H**) enhanced necrosis over 24 h. Two-way ANOVA with Bonferroni’s post hoc test (*n* = 3). * *p* ≤ 0.05, ** *p* ≤ 0.01, *** *p* ≤ 0.001, **** *p* ≤ 0.0001.

We then compared rates of survival between wild-type and Pink1 knockout cells. We found Pink1-KO were significantly more difficult to grow in culture, with less than half the plating efficiency of WT cells (Figure 4A). Nevertheless, those Pink1-KO cells that did adhere appeared to proliferative more robustly than WT cells (Figure 4B). Initial rates of apoptosis and necrosis far exceeded WT cells and suggest Pink1-KO induced a highly unstable cell phenotype. To assess this phenotype further, we assessed oxidative and glycolytic switching by incubating with this cell line with Klotho (Figure 5A–D)) We observed that Klotho reduced reliance on oxidative metabolism (Figure 5A,B) and reduced the ability of Pink1-KO cells to use glycolysis after a glucose bolus (Figure 5C,D). We found Klotho protected initial cell number (Figure 5E) from increased proliferation (Figure 5F), while the rate of cell clearance from apoptosis (Figure 5G) and necrosis (Figure 5H) was markedly improved.

## 3. Discussion

Improving vascular smooth muscle cell longevity is an important though challenging goal of delaying features of vulnerable or rupture-prone atherosclerosis. Indeed, VSMCs provide biochemical and mechanical strength to the plaque through the production of collagen and extracellular matrix, which are features lost as the plaque ages.

Previously, we identified loss of mitochondrial homeostasis as key to this phenotype in human atherosclerosis [1]. More recently, we showed that a switch to glycolytic metabolism as a potential survival mechanism could delay these effects [15]. Here, we undertook a novel drug screen that investigated the role of compounds previously identified as having an energetic switching role through AMPK and are predicted to positively impact energy balance. As previously mentioned, we assessed a drug-specific chronic 24 h incubation or 2 h acute responses based on the previous literature and to mitigate off-target effects. For example, losartan is a selective angiotensin II (Ang II) type 1 (AT(1)) receptor antagonist that has been shown to activate AMPK, and its downstream target acetyl–Co-A carboxylase to mediate effects on the VSMC cell cycle [17]. Berberine has been shown to transactivate AMPK and upregulate expression mitochondrial uncoupling protein UCP1 in which the authors showed evidence of reduced oxidative stress and vascular inflammation [18]. Salicylate has been shown to bind the same sites as A-769662 to improve lipid homeostasis in isolated cells and animal models via activation of AMPK [19,20]. As there is abundant evidence of the role of the drug candidates selected, these were summarised, tabulated, and referenced for ease (Supplemental Table S1 [21,22,23,24,25,26,27,28,29,30,31,32]).

Despite AMPK being the proposed target for the action of these drugs, we found a wide variety of responses and therapeutic doses, ranging from mM doses for metformin to sub nM concentrations for Klotho. This broad range of responses makes interpretation and comparison particularly difficult. Therefore, we chose a more direct measure of energetic performance by measuring oxidative and glycolytic capacity. The XF24 method allows simultaneous readings of both oxygen consumption as an index of mitochondrial energetic performance in real time, as well as changes in milli-pH, to reflect rates of glycolysis. Across a range of nM doses, we validated Klotho as the most impressive candidate that suppressed VSMC oxidative metabolism and induced a reciprocal effect in enhancing glycolytic metabolism. Of note was the reverse dose–response, in which increasingly lower concentrations of Klotho had a more significant impact on oxygen consumption. This may reflect the lower and near physiological dosing of klotho.

To identify if these changes in metabolism influenced cell phenotype, we first compared primary Pink1 wild-type VSMC to Klotho. We observed no significant difference in cell survival between wild-type cells in the presence or absence of Klotho, confirming that Klotho does not directly influence plating efficiency or total cell survival over 24 h in culture. However, wild-type cells incubated with Klotho (0.01 ng/mL) increased their rate of proliferation which was significant at 12 h. The noticeable trend of increased apoptosis and necrosis over 18–24 h could then be advantageous if it reflected Klotho’s restoration of clearance of defective cell types found in the plaque.

Next, we compared Pink wildtype and Pink1-KO cell lines, both derived from ApoE knockout mice. Pink1 kinase KO was chosen as a comparator, as it has previously been shown to be unable to maintain mitochondrial homeostasis and thereby mimic some features of those found in diseased human plaque VSMCs. Using live-cell imagi-ng, we assessed survival, proliferation, apoptosis, and necrosis as an index of drug responses. Pink1-KO cells were more difficult to culture and had ~50% reduced viability from the outset. This effect appeared to be compensated by a higher rate of proliferation but also higher rates of apoptosis and necrosis. These data highlighted the severity of the defect of Pink1-KO and that failure to maintain mitochondrial homeostasis was adversely affecting the cell’s ability to be maintained in culture. Notably, we found Pink1-KO cells had an accumulation of intracellular lipid vesicles. Long-term use of hydroxytamoxifen (OHT) in vitro (the metabolised form of tamoxifen) required to induce gene knockout can promote a more lipogenic phenotype; however, our transient use in vitro for Cre activation (<24 h) is unlikely to be sufficient for this effect. It is intriguing, though highly speculative, that loss of β-oxidation and loss of lipid processing at the mitochondria may be the result of the accumulation of these lipids, but further research is required to explore these observations. Lastly, we tested if this unstable Pink1-KO phenotype could be rescued by Klotho, according to which we had shown WT VSMC can switch between oxidative and glycolytic metabolism. The addition of Klotho appeared to promote survival but block any further proliferation driving a significant increase in both apoptosis (*p* = 0.05) and necrosis (*p* = 0.001). These data suggest that VSMCs are heavily reliant on mitochondrial metabolism for survival, and any switch to a more glycolytic phenotype is unable to easily rescue this type of Pink1-KO induced dysfunction. However, perhaps in the absence of Pink1 which flags mitochondria for degradation, Klotho may compensate, promoting cell clearance through apoptosis or necrosis. We and others have shown that mitochondrial respiratory proteins activity is significantly affected in the most vulnerable parts of the plaque [1,33]. The antiageing hormone Klotho decreases as we age, and research has found it acts in several ways to delay normal human ageing at the cellular and mitochondrial levels, but exactly how remains unknown. For example, Klotho is required for inner mitochondrial membrane super complex formation that reduces aberrant respiratory chain generation of reactive oxygen species. Previous studies have demonstrated that Klotho increases wound healing, potentially through improving stem cell activation and by reducing lineage conversion via DNMT3a and H3K9me2 methylation patterns at critical gene loci [34]. Wnt, TGFβ and FGF signalling, and hexokinase II (Mt-HexII) have also been demonstrated to emanate from the mitochondria in response to energetic decline that may promote necrosis which may explain our observations in Pink1-KO VSMCs [35].

## 4. Materials and Methods

### 4.1. Generation of VSMC Wild-Type and Pink1 Cell Lines

Explant cell lines were generated from excised whole murine aorta from ApoE and Pink1 ApoE mouse colonies, hereby termed VSMCs. Briefly, mice were humanely culled and aortic tissues harvested and washed in PBS. Aortae were immediately cleaned of extraneous blood and perivascular fat. The vessels were dissected in 1 mm^2^ cubes and placed on a wetted 25 cm^2^ flask containing 5 mL of DMEM media supplemented with 10% FCS. Flasks were left undisturbed for 7 days in humidified 5% CO_2_ incubator at 37 °C. At ~10% confluency flasks were trypsinised and reseeded into the same flask to promote isolated cell growth. Flasks were then subcultured and frozen 1 × 10^6^ cells/mL in 10% DMSO for long-term storage. In addition to the ApoEs colonies, Pink1 mouse colonies had previously confirmed gene knockout by Southern blotting, RT-PCR [36] and were routinely DNA genotyped by PCR. In addition, a Cre responsive X-gal reporter in the ROSA26 locus was used as confirmation. Briefly, tissues were harvested, washed in PBS, and mounted in OCT compound and frozen sections cut at 5 µm intervals. Pink1 cells were treated with a single low dose hydroxytamoxifen (OHT) 100 nM for a maximum of 48 h (Sigma-Aldrich 68047-06-3, St. Louis, MO, USA) and then washed in 1xPBS and cultured or fixed at room temperature for 5 min in 4% paraformaldehyde, supplemented with 0.1 N Sodium PO_4_. Specimens were incubated overnight in 1 mL of staining buffer: 200 mM MgCl_2_, 400 mM ferricyanide, 400 mM ferrocyanide, X-gal solution in PBS, then briefly washed in MQ and visualised.

### 4.2. MTT Viability Assay

Drug cytotoxicity and dose–response curves were performed using a standard MTT cell viability assay [37]. Primary VSMC were plated at 7000 cells/well in a 96-well plate. Drug incubation was established for 2–24 h incubation (37 °C with 95% oxygen and 5% CO_2_) with a serial drug dilution in DMEM. After each drug incubation period, a further 6 h incubation with 50 μL of 5 mg/mL MTT Formazan (Sigma Aldrich 57360-69-7) was added to each well, wrapped in foil, and incubated in the same way. At the end of the MTT incubation period, all media was replaced with 100 μL of 99.5% dimethyl sulphoxide (DMSO) and 50 μL of glycine buffer (0.1 M glycine and 0.1 M NaCl adjusted to 10.5 pH with 1 M NaOH). The plate’s absorbance was immediately read at 560 nm using a Victor^TM^ X3 PerkinElmer plate reader (PerkinElmer, Waltham, MA, USA).

### 4.3. Oxygen Respirometry

The XF24 Seahorse assay (Agilent Technologies, Santa Clara, CA, USA) was used for cell-based mitochondrial and glycolytic determination. Primary explanted murine VSMCs were seeded into a microplate with 100 μL at 15,000 cells/well. A calibration plate was prepared with 0.5 mL of XF calibrant solution. Cell plates media was exchanged with 0% serum-free Seahorse media on the day of the assay with a minimum of five technical replicates per plate were taken. All injection ports were prepared as previously described [1].

### 4.4. Live-Cell Scoring Analysis

Bright-field images and scratch assay were captured and scored using Olympus IX73 and BX51 microscopes using Adobe Photoshop^TM^, ImageJ, and Olympus CellSenS software, Southend-on-Sea, United Kingdomas previously described [15,38].

### 4.5. Western Blotting

Western blots were performed as previously described [39,40,41].

### 4.6. Statistical Analysis

All data are presented as mean ± standard error of the mean (SEM). Significance values were calculated using Student’s paired *t*-tests (two-tailed distribution) for data following a Gaussian distribution for normality. Statistical significance is presented as *p* < 0.05 (one star), *p* < 0.01 (two stars) or *p* < 0.001 (three stars) *p* < 0.0001 (four stars). For multiple comparisons, a one-way analysis of variance (ANOVA) was used to determine a significant difference between two or more independent groups with Dunnett’s post hoc analysis. For two-way multiple comparisons, a two-way ANOVA was used with a Bonferroni post hoc test. All analysis was performed and plotted on GraphPad Prism (V. 9.3.1), GraphPad Software, San Diego, CA, USA.

## 5. Conclusions 

In this study, we predicted that VSMCs would use a mix of mitochondrial and glycolytic metabolism, but as the plaque age, mitochondrial dysfunction increases. We found Klotho could have a significant role in improving VSMC phenotype, and targeting Klotho at earlier time points in disease could induce energetic switching and improve VSMC survival in the plaque. Even modest improvement in survival and proliferation would strengthen the plaque cap and could theoretically delay rupture. Using a VSMC specific Pink1-KO, we identified a highly unstable cell line in which Klotho was able to enhance cell clearance which would be predicted to be beneficial by removing defective cell types found in the plaque. Rewiring glycolysis has been attempted before, using embryonic fibroblasts and the transcription factor Lin28 in both wild-type and Pink1-KO cell lines, with limited efficacy [42,43]. Our data now provide a novel cell-based methodology in which candidate drugs can be screened and assessed for suitability. It also suggests that Klotho may play a more direct role in mitochondrial function, improve wound response and may prove useful if targeted to plaque vascular cells in vivo.

## Data Availability

Supporting data can be made available on request.

## Supplementary Materials

The following are available online at https://www.mdpi.com/article/10.3390/ijms23010217/s1.
