# Supplementary material for: Inducing Energetic Switching Using Klotho Improves Vascular Smooth Muscle Cell Phenotype
Source: Int J Mol Sci. 2021 Dec 25;23(1):217. doi: 10.3390/ijms23010217 (PMC8745077; doi:10.3390/ijms23010217)
Supplement: Supplementary file 1 [file ijms-23-00217-s001.zip › ijms-1512100-SI.pdf]

Supplemental Figures

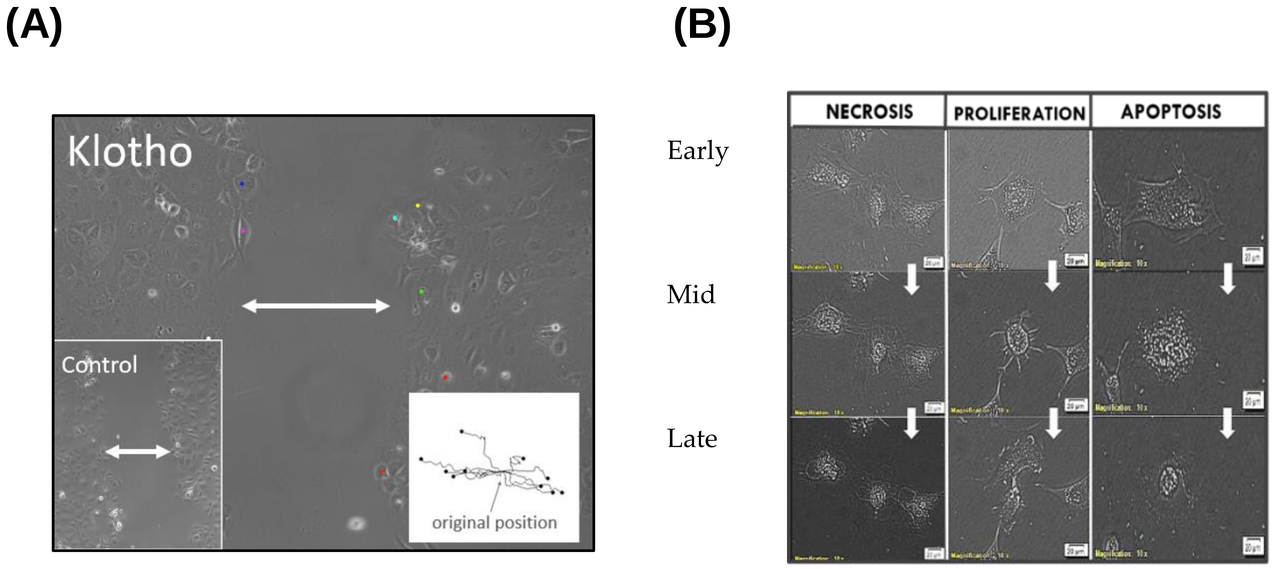

Supplemental Figure S1. *Live cell image of phenotypic changes oin VSMC in the presence and absence of Klotho (**A**) Live cell time-lapse imaging following scratch assay closure with improved migration of klotho treated monolayers over 24 hrs (n=3),(Image-J cell tracke module). (**B**) Representative images tracking the different live cell phenotypes over time (white arrows show progression from top to bottom images) for necrosis, mitosis and apoptosis.*

Supplemental Table S1. List of drugs screened for changes in cell energetics.

Compound	Doses	Stimulation (hr)	Mechanism	References
**AICAR (in H_2O)**	0.1; 0.25; 0.5; 1; 2 mM	2 h	AMP mimetic	Goirand et al 2007, [21] Sun et al 2007,[22]
**A-769662 (in DMSO)**	5; 10; 20; 50; 100 μM	2 h	Allosteric activation of AMPK (Acting on AMPKβ1)	Treebak 2009, [23] Goransson, 2007[24]
**Berberine (in DMSO)**	10; 20; 50; 100; 200 μM	24 h	Complex 1 inhibition	Wang et al, 2011[18] Lee et al 2006[25]
**Etoposide (in DMSO)**	5; 10; 20; 50; 100 μM	24 h	ATM-dependent mitochondrial biogenesis and AMPK activation	Fu et al, 2008[26]
**Klotho (in H_2O) ([1]membrane[2]soluble[3]intracellular)**	1.0 to 0.0001ng//mL	2-24 h	1) FGF23 co-receptor<br>2) Inhibitor of IGF/TGF-β1<br>3) Cytokine suppressant & antioxidant	Wolf I 2008[11] Imura et al 2007 [12] Kurosu et al 2005[7]
**Losartan (in H_2O)**	1; 2.5; 5; 20; 50 μM	24 h	p21 and p53 expression	Kim et al 2010[17]
**Metformin (in H_2O)**	0.1; 0.5; 1.0; 5; 10 mM	2 h	Complex 1 inhibition	Zho et al[27]
**Resveratrol (in DMSO)**	10; 20; 50; 70; 100 μM	24 h	SIRT1-dependent mitochondrial biogenesis	Price et al, 2012[28]
**Trimetazidine (in H_2O)**	0.1;1.0;10;100 μM	2-24 h	Inhibitor of free fatty acid oxidation and increased glucose utilisation	Zheng, S. *et al* 2018[29] Chrusciel et al 2014[30] Marzilli et al 2019[31]
**Troglitazone (in DMSO)**	5; 10; 20; 50; 100 μM	24h	Mitochondrial membrane depolarisation	Le Brasseur et al, 2006[32]
**Salicylate (in H_2O)**	0.5; 1; 2; 5; 10 mM	24h	Allosteric activation of AMPK	Hawley et al, 2012[20]

Supplemental Table S2. Primary and secondary antibodies used in immunocytochemistry and immunohistochemistry. (All others as previously described Mercer *et al* 2005).

Pathway	Antibody	ICC 1 ⁰Ab	IHC 1⁰Ab	Company	Secondary antibody
**p-ACC 280kDa (Cyto and SR)**	Phospho-Acetyl-CoA Carboxylase (Ser 79) Antibody #11818 (inhibitory side phosphorylation)	1:100	1:200	Cell Signalling Technology ©	Anti-Rabbit Alexa543 1:500
**Total ACC 280kDa**	ACC Antibody #3676 Cyto and SR) (not shown)	1:250	1:400	Cell Signalling Technology ©	Anti-Rabbit Alexa543 1:500
**α-SM-Actin Cytoplasm)**	α-SM-Actin Antibody (Mouse)	1:100	1:200	Abcam ©	Anti-Mouse (alexa543) 1:500
**AMPK 62kDa (Cytoplasm)**	Phospho-AMPKa (Thr172) Antibody (Rb) #2535	1:100	1:200	Abcam ©	IgG Anti-Mouse 1:500
**Phospo-Pink1**	Ab117091 Ab237070	1:100	1:200	Abcam©	IgG Donkey Anti-rabbit Ab150073 and Ab175780 RP
